# Isolation and Selection of Duck Primary Cells as Pathogenic and Innate Immunologic Cell Models for Duck Plague Virus

**DOI:** 10.3389/fimmu.2019.03131

**Published:** 2020-01-28

**Authors:** Bin Tian, Dongjie Cai, Tianqiong He, Liyao Deng, Liping Wu, Mingshu Wang, Renyong Jia, Dekang Zhu, Mafeng Liu, Qiao Yang, Ying Wu, Xinxin Zhao, Shun Chen, Shaqiu Zhang, Juan Huang, Xumin Ou, Sai Mao, Yanling Yu, Ling Zhang, Yunya Liu, Anchun Cheng

**Affiliations:** ^1^Institute of Preventive Veterinary Medicine, Sichuan Agricultural University, Chengdu, China; ^2^Research Center of Avian Disease, College of Veterinary Medicine, Sichuan Agricultural University, Chengdu, China; ^3^Key Laboratory of Animal Disease and Human Health of Sichuan Province, Sichuan Agricultural University, Chengdu, China

**Keywords:** duck primary cell isolation, duck plague virus, innate immune induction, IFNAR signaling, abortive infection

## Abstract

Duck plague virus (DPV) is a representative pathogen transmitted among aquatic animals that causes gross lesions and immune inhibition in geese and ducks. The mechanism of organ tropism and innate immune evasion of DPV has not been completely deciphered due to a lack of cell models to study the innate immune manipulation and pathogenicity of aquatic viruses. In the present study, we isolated five types of duck primary cells [duck embryo fibroblasts (DEFs), neurons, astrocytes, peripheral blood mononuclear cells (PBMCs), and monocytes/macrophages] to identify appropriate cell models for DPV, using tropism infection and innate immunologic assays. Cells responded differently to stimulation with DNA viruses or RNA virus analogs. DPV infection exhibited broad tropism, as the recombinant virulent strain (CHv-GFP) infected DEFs, neurons, astrocytes, and monocytes/macrophages, but not the PBMCs, as the expression of EGFP was negligible. The basal levels of innate immunity molecules were highest in monocytes/macrophages and lower in DEFs and astrocytes. Conversely, the titer and genomic copy number of the attenuated virus strain was higher in DEFs and astrocytes than in neurons and monocytes/macrophages. The titer and genomic copy number of the attenuated virus strain were higher compared with the virulent strain in DEFs, neurons, and astrocytes. The innate immune response was not significantly induced by either DPV strain in DEFs, neurons, or astrocytes. The virulent strain persistently infected monocytes/macrophages, but the attenuated strain did so abortively, and this was accompanied by the phenomenon of innate immune inhibition and activation by the virulent and attenuated strains, respectively. Blockage of IFNAR signaling promoted replication of the attenuated strain. Pre-activation of IFNAR signaling inhibited infection by the virulent strain. The selection assay results indicated that induction of innate immunity plays an essential role in controlling DPV infection, and monocytes/macrophages are an important cell model for further investigations. Our study provided practical methods for isolating and culturing duck primary cells, and our results will facilitate further investigations of organ tropism, innate immune responses, latent infection, and the effectiveness of antiviral drugs for treating DPV and potentially other aerial bird pathogens.

## Introduction

The innate immune response is the first line of host defense against microbial pathogens. Various pattern recognition receptors (PRRs) play an essential role in detecting pathogen-associated molecular pattern (PAMP) associated with invading pathogens. PAMP recognition initiates an innate immune response, characterized by the production of type I interferon (IFN), proinflammatory cytokines, and IFN-stimulated genes (ISGs) (1, 2). PRRs comprise multiple family members, including toll-like receptors (TLRs), retinoic acid inducible gene I (RIG-I)-like receptors (RLRs), nucleotide oligomerization domain (NOD)-like receptors, C-type lectin receptors, and cytosolic dsDNA sensors (CDSs). RLRs primarily recognize 5′-phoshorylated RNAs, including ssRNA or dsRNA produced during the replication of RNA or DNA viruses (3). Although RIG-I predominantly recognizes RNA viruses, the RIG-I/Mitochondrial antiviral-signaling protein (MAVS) pathway is also activated during infection with several DNA viruses, including herpes simplex virus (HSV-1), Epstein-Barr virus, and Kaposi's sarcoma-associated herpesvirus (4–6). During infection with DNA viruses, RNA polymerase III recognizes AT-rich dsDNA and transcribes the dsDNA into dsRNA containing a 5-triphosphate moiety that activates the RIG-I/MAVS pathway to induce IFN-β production (7). DNA from viruses or bacteria can be detected by Cyclic GMP-AMP synthase (cGAS) and potentially RLR, which potentially activate the endoplasmic reticulum-resident adaptor protein, stimulator of interferon genes (STING), to translocate from the endoplasmic reticulum to the Golgi, where it activates TBK1-IRF3 and -NF-κB, resulting in robust induction of type I IFN and inflammatory cytokine production (8). IFN-I binds to IFNAR and activates R1-associated Tyk2 protein tyrosine kinase and the IFN-alpha/beta R2-associated Jak1 protein tyrosine kinase, which subsequently regulate the phosphorylation and activation of different STAT proteins; the activated STAT proteins homo- or heterodimerize and translocate to the nucleus, where they promote the expression of numerous target genes. Binding of STAT proteins to either ISREs or GAS sites regulates the expression of several hundred ISGs, which mediate the anti-viral, anti-proliferative, and apoptotic effects of type I IFNs.

Duck plague (DP), also known as duck viral enteritis (DVE), is caused by Anatid herpesvirus type 1 (AHV-1) or duck plague virus (DPV), which is an enveloped, dsDNA virus of the *Herpesviridae* family, subfamily *alpha-Herpesvirinae* (9, 10). *First* reported in the Netherlands in 1923, DP spread rapidly around the world (11, 12). Although typically an acute or sometimes chronic and highly contagious disease, DP is characterized by high mortality rates (up to 100%) among domestic (12) and wild ducks, swans, geese, and other waterfowl of different ages. To prevent DP outbreaks on duck farms, attenuated DPV vaccines have been widely used; in China, use of these vaccines is compulsory, with billions of doses administered annually (13, 14).

DPV is the only herpes virus circulating in aquatic animals identified to date. Infection with virulent DPV strains causes gross lesions in ducks in most tissues, including the heart, liver, spleen, bursa, and brain (15, 16), where the virus has been detected (12, 17). Upregulation of PRRs and ISGs expression has been reported, indicating that DPV exhibits broad organ tropism and activates the innate immune system (18, 19). Differing basal and induced levels of PRRs and ISGs among different cell types and organs are important factors in determining the organ tropism of viruses such as poliovirus, reovirus, and murine coronavirus (20–22). Recently published data indicated that expression of RIG-I, galectin-1, MAVS, STING, and IRF1 is induced in DPV-infected ducks, demonstrating the strong capacity of the innate immune response to restrict DPV infection via over-expression of these factors in DEFs, although it is difficult to detect changes in these factors in DEFs infected with a high titer of DPV (23–27).

According to a previous study, TLR8, IRF3, ISG15, ISG54, and ISG56 (IFITs) are missing in birds, chickens also lack RIG-I and Riplet (28), and the immune system of birds is different from that of mammals. Development of a suitable cell model for in-depth investigations of the mechanism of the innate immune response to DPV and the virus's ability to evade that response is thus an important priority. In the present study, therefore, we isolated and cultured five types of duck primary cells *in vitro* and then compared the basal and innate immune responses to DNA and RNA virus analogs. The cell tropism of DPV and changes in innate immune signaling induced by DPV infection and the antiviral effect of IFNAR signaling against DPV infection were also investigated. The isolation and characterization of different types of duck primary cells could facilitate elucidation of the mechanism governing the organ tropism of DPV and the relationship between DPV infection and host antiviral innate immune responses.

## Materials and Methods

### Ethics Statement

All animal experiments were conducted in accordance with approved guidelines. One-month-old Peking ducklings were purchased from a DPV-free farm where vaccination against DPV was not implementation. All the ducks were housed in the animal facility at Sichuan Agricultural University, Chengdu, China. The study was approved by the Committee of Experiment Operational Guidelines and Animal Welfare of Sichuan Agricultural University (approved permit number XF2014-18).

### Duck Embryo Fibroblast Isolation and Culture

Nine-day-old duck embryos were cleaned with 75% ethanol and placed on a 6-well plate. The head, wings, legs, and viscera were removed, and the muscle tissues were washed with HBSS, cut into 1-mm pieces, and then digested with 0.1% trypsin for 10 min at room temperature (RT). After digestion, the trypsin was removed via centrifugation at 5,000 rpm for 5 min, and the digested tissues were dissociated by repeated pipetting (~30 times), after which the mixture was filtered through autoclaved medical gauze. Single cells were collected and plated in cell culture plates or dishes and cultured in MEM supplemented with 10% fetal bovine serum (FBS; Gibco-BRL, Carlsbad CA, USA). Usually, DEFs were cultured to 100% confluence for 24 h and then passaged and sub-cultured for use in subsequent assays.

### Isolation of Duck Neurons

Duck neurons were isolated according to our previously described method for isolating mouse neurons (29). Briefly, the brain was collected from 9-day-old duck embryos, the meninges were peeled away and carefully removed, and the cortex was transferred into a new dish filled with HBSS, and then cut into 1-mm pieces by using scissors; the shears were transferred into 0.1% trypsin diluted in HBSS and digested for 20 min at RT. The trypsin was removed by transferring the brain cells into a new tube with proper DMEM, and then DNase I was added and treated for 5 min at RT. The DNase I was removed and the cells were collected by centrifugation at 1,200 rpm for 10 min at RT. Then, the collected cells were resuspended with DMEM and dissociated by repeated pipetting (<15 times). The cells were passed through a 75-nm nylon mesh (Corning, NY, USA) to separate the single cells, washed once in HBSS, and then cultured in D-polylysine–pretreated plates in DMEM supplemented with 5% FBS and 1% penicillin–streptomycin for 6 h. Finally, the cells were washed once with phosphate-buffered saline (PBS), and the medium was replaced with serum-free, neural-basal medium supplemented with 2% B-27 PLUS (Gibco-BRL, NY, USA), and the cells were incubated for 5 days to form a monolayer.

### Isolation of Duck Primary Astrocytes

Primary duck astrocytes were isolated primarily according to the procedure described above for isolating duck neurons, with some differences. Brain cells passed through a 75-nm nylon mesh were cultured in DMEM supplemented with 10% FBS and 1% penicillin–streptomycin for 24 h, washed once with PBS, and then cultured in the same medium for 4 days to form a monolayer. The medium was changed every 2 days. Similar to DEFs, astrocytes readily agglomerated and detached from the plates or dishes once they began to overgrow.

### Isolation of Duck PBMCs

Duck PBMCs were isolated from the 1-month-old anti-coagulation whole blood by density gradient centrifugation using a Duck Leukocyte Isolation kit (TBDscience, Tianjin, China), according to the manufacturer's instructions and our previous study (30). Briefly, whole blood was collected from the jugular vein of mature ducks and placed in anticoagulant-containing tubes. The blood was then diluted with sample dilution buffer and slowly added onto a layer of duck lymphocyte isolation buffer (density: 1.077 ± 0.001 g/ml) to avoid mixing and then centrifuged at 500 g for 30 min at RT. The second grayish-white layer was transferred into a new tube, and resuspended in 20 ml washing buffer and the mixed cells were collected by centrifugation at 500 g for 10 min at RT. The collected cells were resuspended in 3 ml of red blood cell lysis buffer for 3 min and then 20 ml of washing buffer was added into the cells. Then, the cells were collected by centrifugation at 500 g for 10 min at RT. The remaining cells were adjusted to 2 × 10^7^ cells/ml and 1 ml of cells was plated in one well of the 12-well plate, and cultured in RPMI 1640 medium supplemented with 10% FBS and 1% penicillin–streptomycin for 2 h to allow for attachment to the plate; unattached cells were removed by washing twice with PBS, followed by addition of fresh medium. The isolated PBMCs were then ready for use in assays.

### Isolation of Duck Monocytes/Macrophages

Duck monocytes/macrophages were isolated according to a reported method for isolating human macrophages (31). Briefly, duck PBMCs were prepared as described above. Adherent cells were enriched among PBMCs by adherence on plastic culture plates for 2 h. Non-adherent cells were removed via vigorous washing three times using pre-warmed PBS; the adherent cells were digested with trypsin for cell count. Duck monocyte-derived macrophages were differentiated from adherent monocytes in RPMI 1640 medium supplemented with L-glutamine (2 mM), sodium pyruvate (1 mM), 10% heat-inactivated FBS, 1% penicillin–streptomycin, and 50 ng/ml human M-CSF (Novoprotein, Shanghai, China). The medium was changed every 2 days, and duck macrophages formed a monolayer by day 7. In our present isolation method, only adherent cells are able to differentiate into macrophages under the induction of M-CSF; lymphocytes in PBMCs (mainly including T cells, B cells, and NK killer cells) continue to die due to inability to differentiate under induction of M-CSF and are removed by constantly replacing fresh medium.

### Antibodies, Virus Strains, and Reagents

The virulent strain of DPV, CHv, was isolated and characterized by our lab (32). The recombinant virulent strain of DPV, BAC-CHv-EGFP (CHv-GFP), was constructed by our research center (33). The attenuated vaccine strain of DPV, CHa, was retrieved from storage at our research center. The Duck Tambusu virus (DTMUV) was stored at our research center. Each DPV and DTMUV strain was propagated on DEFs. The Rabbit anti-human MAP2 and -human GFAP polyclonal antibody and the goat anti-human β-actin monoclonal antibody were purchased from Abclonal (Wuhan, China). Mouse anti-duck CD80 (Gene ID:101796029) and CD86 (Gene ID:101794331) antibodies were generated by our research center. Ruxolitinib, poly(dA:dT), and poly(I:C) were purchased from Invivogen (Hong Kong, China).

### Western Blot

The cells were lysed with RIPA buffer (50 mM Tris, pH 8.0; 150 mM sodium chloride; 1% Triton X-100; 0.5% sodium deoxycholate; 0.1% SDS) containing protease inhibitors (Roche), and the protein concentrations were measured using a DC protein assay kit (Bio-Rad). Equal quantities of protein were resolved by 12% SDS-PAGE and then transferred to polyvinylidene difluoride (PVDF) membranes (Bio-Rad), which were blocked with 5% non-fat milk before being incubated with primary antibodies against CD80, CD86, or β-actin and then probed with the appropriate secondary antibodies. The blots were then visualized using ECL reagent (GE, Pittsburgh, PA, USA) and detected under an Intelligent dark box II (GE, Pittsburgh, PA, USA).

### Innate Immune Response of Duck Primary Cells to DNA and RNA Virus Analogs

Duck primary DEFs, neurons, astrocytes, PBMCs, and monocytes/macrophages were treated with the DNA and RNA virus analogs poly(dA:dT) or poly(I:C), respectively, at a dose of 5 μg/ml for 24 h. The cells were then lysed in Trizol reagent for RNA isolation to assess the innate immune response to the stimulators using quantitative real-time polymerase chain reaction (qRT-PCR).

### Indirect Immunofluorescence Assay (IFA)

Primary duck neurons, astrocytes, or monocytes/macrophages were cultured for 4, 4, or 7 days, respectively, to form a confluent monolayer. The cells were then fixed with 4% neutral buffered paraformaldehyde for 20 min, permeabilized with 0.2% Triton X-100 for 10 min, blocked with 10% BSA dissolved in PBS for 30 min, and then incubated with primary antibodies against MAP2, GFAP, CD80, and CD86 at 4°C overnight. The cells were incubated with Alexa Fluor 488-conjugated goat anti-rabbit or -mouse secondary antibodies or Alexa Fluor 594-conjugated goat anti-rabbit or -mouse secondary antibodies for 1 h at RT. Images were acquired using fluorescence microscopy.

### Virus Infection and Determination of TCID_50_

For virus infection, the required dose of virus was diluted in the medium used to culture the various types of duck primary cells (5 × 10^6^ cells in a 12-well plate) and incubated with cells at 37°C for 1 h. The cells were then washed twice with PBS and maintained in the corresponding medium supplemented with 2% FBS and 1% penicillin–streptomycin. The culture supernatant of virus-infected cells was collected and titrated to determine the tissue culture infectious dose 50 (TCID_50_) on DEFs using 10-fold serial dilutions.

### Determination of Virus Copy Number

Viral DNA was extracted using a HIPURE viral DNA mini kit (Magen, Guangdong, China) according to the instructions provided by the manufacturer. DPV genomic DNA in infected cells was quantified using an absolute Q-PCR method as previously described (17) using primers specific to the sequence of DPV UL30 (primers are listed in Table 1). A standard curve was generated from serially diluted plasmids harboring the entire Coding sequence of UL30 and using the same PCR procedure as used for cell samples. DPV copy number in infected cells was calculated according to the standard curve and normalized to 1 μg of total DNA.

**Table 1:** Primers used for quantification of PRR, IFN, ISG, and cytokine mRNAs and viral genomic DNA.

**Primer name**	**Accession no**.	**Nucleotide sequence (5^**′**^-3^**′**^)**	**Use**
cGAS F	XM_021271479.1	CCCTACCACCAATGTCACCC	qRT-PCR
cGAS R		GGTTGCACTGGGGAGATTCA	qRT-PCR
STING F	XM_021273408.1	CCACATCTTGATCCCGCTGA	qRT-PCR
STING R		ATTGCGTAGAGGCTGTGCTT	qRT-PCR
RIG-I F	KC869660.1	TGAGCTGCAAGGGAGACAAG	qRT-PCR
RIG-I R		TCCAATTCAGCTGACAGGGC	qRT-PCR
MDA5 F	KJ451070.1	GCTGAAGAAGGCCTGGACAT	qRT-PCR
MDA5 R		TCCTCTGGACACGCTGAATG	qRT-PCR
IRF7 F	MG707077.1	AACATCTCCAACACCGACCC	qRT-PCR
IRF7 R		CTCCTGGGATGGTTTGCTCC	qRT-PCR
IFN-β F	KM035791.2	TCTACAGAGCCTTGCCTGCAT	qRT-PCR
IFN-β R		TGTCGGTGTCCAAAAGGATGT	qRT-PCR
MX F	NM_001310409.1	TGCTGTCCTTCATGACTTCG	qRT-PCR
MX R		GCTTTGCTGAGCCGATTAAC	qRT-PCR
IL-6 F	XM_013100522	TTCGACGAGGAGAAATGCTT	qRT-PCR
IL-6 R		CCTTATCGTCGTTGCCAGAT	qRT-PCR
18S F	AF173614.1	TGTGCCGCTAGAGGTGAAATT	qRT-PCR
18S R		TGGCAAATGCTTTCGCTTT	qRT-PCR
β-actin F	EF667345.1	GCCCTCTTCCAGCCATCTTT	qRT-PCR
β-actin R		CTTCTGCATCCTGTCAGCGA	qRT-PCR
TLR2 F	KX687002.1	AAAACGCTCTTCGTGCTGTC	qRT-PCR
TLR2 R		CTCCTGCTGCTCTTCCTCAC	qRT-PCR
TLR4 F	JN618073.1	AGTTTGACATTGCCCAGTCC	qRT-PCR
TLR4 R		TCCTCCTCGTGATTCCATTT	qRT-PCR
CD80 F	XM_005017637.3	GCCCCTCATCAATGGTCACA	qRT-PCR
CD80 R		CCCCACCCATTATCCCACAC	qRT-PCR
CD86 F	XM_027449711.1	GGCCCGAGGTCCCATAGTAT	qRT-PCR
CD86 R		GAAGACTGAGGAGAGCACTGG	qRT-PCR
DPV UL30 F	JQ647509.1	TTTCCTCCTCCTCGCTGAGTG	Absolute RT-PCR
DPV UL30 R		CCAGAAACATACTGTGAGAGT	Absolute RT-PCR
Taqman probe to DPV UL30		CGCTTGTACCCAGGG	Absolute RT-PCR

### RNA Isolation and qRT-PCR

RNA was extracted from cells using TRIzol® Reagent (Invitrogen) according to the manufacturer's instructions; the genomic DNA was removed and cDNA was synthesized by using NovoScript® Plus All-in-one 1st Strand cDNA Synthesis SuperMix (gDNA Purge) (Novoprotein, Shanghai, China), and qRT-PCR analysis was performed as described previously (29). Briefly, 1 μg of RNA from cells was transcribed into cDNA according to the instructions of the Superscript III Reverse Transcription kit. A total of 1 μl of cDNA was mixed with 5 μl of iQ5 SYBR Green Mix (BioRad, Hercules, CA, USA), 3 μl of double-distilled water, and 0.5 μl each of forward and reverse primer. The cDNA was amplified and the cycle threshold (Cq) values were recorded. The cDNA concentration, primer sequence, and Q-PCR procedure applied for each gene in each cell type were the same. The basal expression level of each gene in each cell type was compared by directly determining from the Cq values according to the method used in the previous study (20). The relative mRNA expression of each gene in each cell type was normalized to the expression level of the 18s mRNA gene (Δ*CT CT* [PRR or ISG]/Δ*CT* [18s]). Expression levels of induced mRNAs are presented as the fold change relative to mock-infection levels according to the 2^−ΔΔ^*CT* method. All primer sequences are listed in Table 1.

### Blockage of IFNAR Signaling

In order to examine the impact of IFNAR signaling on the DPV replication in each cell type, the IFNAR-specific inhibitor, ruxolitinib, was applied. The cell toxicity of ruxolitinib at a dose of 1, 5, 10, and 20 μM/ml on each cell type was detected by MTT method. Duck DEFs, neurons, astrocytes, PBMCs, and monocytes/macrophages were pretreated with 5 μM/ml of ruxolitinib for 1 h and then infected with CHv or CHa at a MOI of 0.1 for 1 h. The cells were then washed twice with PBS, and the same concentration of ruxolitinib was added to the culture medium; DMSO was used as a mock control. The cell culture supernatant in each cell type was collected to detect the TCID_50_ as above described. The cell was scraped in PBS from the plate to detect the viral genomic copy number.

### Antiviral Assay

For the preventative antiviral assay, duck monocytes/macrophages were pretreated with 20 μg/ml poly(dA:dT) or poly(I:C) for 12 h, sterile water was used as control, and then infected with CHv or CHa at a MOI of 0.1. The cells were then washed twice with PBS, and the same concentration of poly(dA:dT) or poly(I:C) was added to the culture medium and incubated for 24 h.

For the therapeutic antiviral assay, duck monocytes/macrophages were pretreated with 10 or 20 μg of poly(dA:dT) or poly(I:C) for 2 h and then infected with CHv or CHa at a MOI of 1.0. After infection, the analogs were added at the same concentration and incubated for 24 h. At 24 hpi, the cell culture supernatants were collected for TCID_50_ determination, and the cells were washed with PBS, scraped from the dishes, and collected for viral genomic copy number determination as described above.

### Statistical Analysis

Data are expressed as the mean and standard error of the mean (SEM), and the significance of differences between groups was evaluated using the Student's *t-*test or one-way analysis of variance followed by Tukey's *post-hoc* test. Asterisks indicate the level of statistical significance (^*^P < 0.05; ^**^P < 0.01; ^***^P < 0.001; ^****^P < 0.00001). All experiments were repeated at least three times individually. Graphs were plotted and analyzed using GraphPad Prism software, version 6.0 (GraphPad Software, La Jolla, CA, USA).

## Results

### Duck Primary Cells Isolation and Characterization

**Figure 1:**
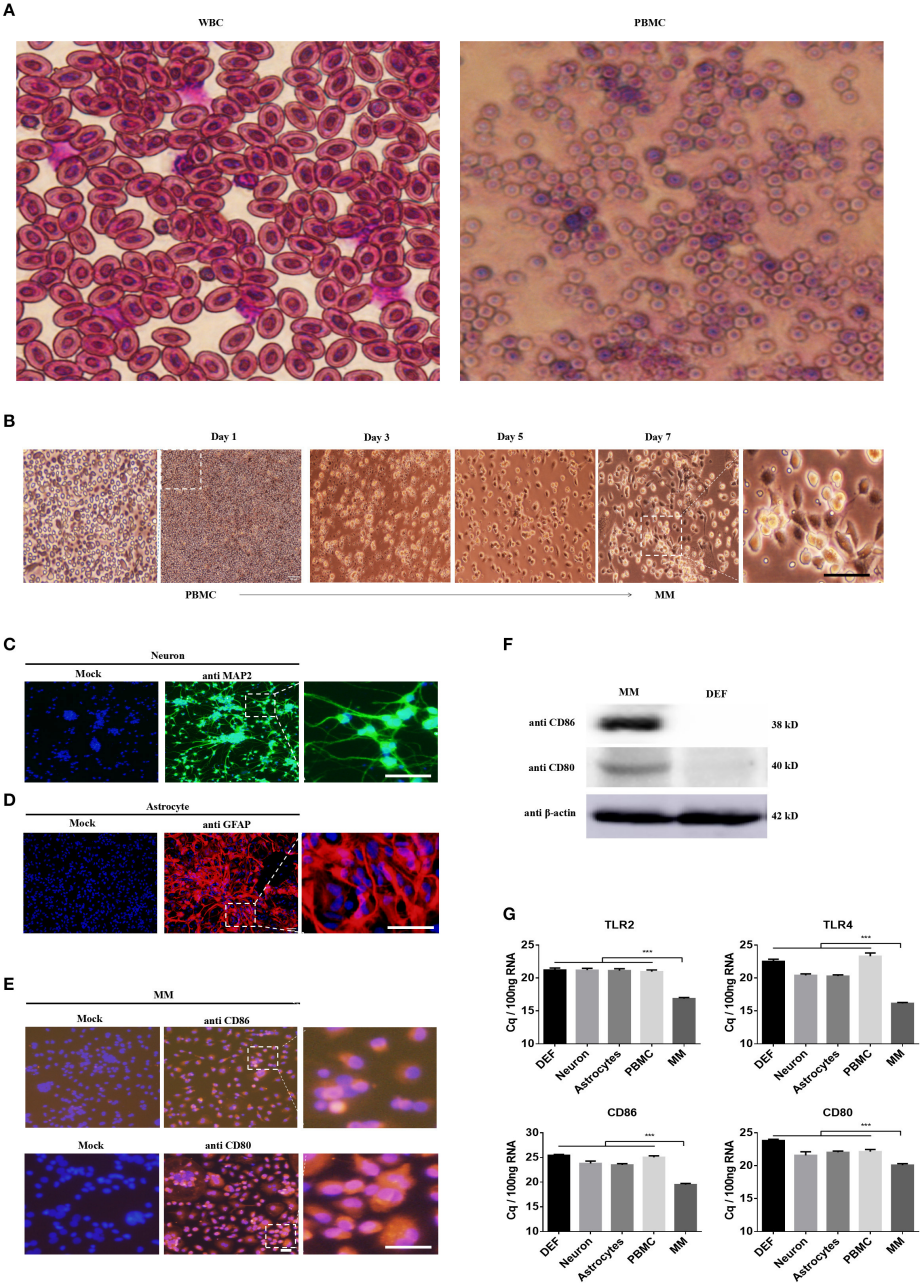
Identification of the neuron, astrocyte, and monocytes/macrophages (MM). **(A)** The PBMCs were isolated from the duck whole blood cells (WBCs); both cells were stained with Wright Strain. At least 15 individual views were observed to calculate the cell populations. **(B)** The time course of PBMCs differentiates into monocytes/macrophages. **(C–E)** Three duck primary cell types were identified via IFA using a rabbit polyclonal antibody against MAP2 for neurons **(C)**, a rabbit polyclonal antibody against GFAP for astrocytes **(D)**, and a mouse polyclonal antibody against CD80 and CD86 for monocytes/macrophages **(E)**. The scale bar is 100 μm. **(F)** The monocytes/macrophages or DEF cells were lysed with RIPA buffer, and the protein expression levels were determined by Western blot using antibodies against CD80, CD80, or β-actin. **(G)** The basal levels of duck TLR2, TLR4, CD86, and CD80 in the five types of duck primary cells were examined using Q-PCR with specific primers. The data are presented as Cq values for 100 ng of RNA.

DEFs have been cultured in our lab using sophisticated methods for many years, and PBMCs were isolated using a duck leukocyte isolation kit; these two cell types were applied in our previous studied (30). Hence, we strove to isolate and culture duck neurons, astrocytes, and monocytes/macrophages according to previous methods used for mouse and human cells. First and foremost, we isolated PBMCs from the duck whole blood cells (WBC) through density gradient centrifugation, the WBCs and PBMCs were stained with Wright Strain (Figure 1A), and the results showed that there are mainly erythrocyte and little partial of thrombocytes and leucocytes in the WBCs. The leucocytes were above 95% in the PBMCs, and the monocytes were about 7% among the total PBMCs (Figure 1A). Since there is no duck-derived M-CSF on sale, we blast the duck's M-CSF protein sequence (31–177aa) with the human's M-CSF protein sequence, and found that the duck's M-CSF protein sequence has the same functional domain as the human M-CSF protein sequence (34–175aa) (data did not show); thus, we tried to stimulate the duck-derived PBMCs with human M-CSF, we observed and collected the time course and found that it can induce duck PBMCs to differentiate into macrophages, which were mainly composed of the wheel and spindle-shaped cells (Figure 1B). In our present method, the survival time of the PBMCs was not more than 72 h without M-CSF, so we believe that the obtained monocytes/macrophages were induced by human M-CSF. Since the antibodies (see below) we used to identify the macrophage-like cells we have obtained were against the surface molecules that are found in both monocytes and macrophages, we term the cells as monocytes/macrophages (MM).

Under specific culture conditions, we successfully cultured duck primary neurons, astrocytes, and monocytes/macrophages and identified the cells using an IFA with a rabbit polyclonal antibody against MAP2 for neurons (Figure 1C), a rabbit polyclonal antibody against GFAP for astrocytes (Figure 1D), and a mouse polyclonal antibody against CD80 and CD86 for monocytes/macrophages (Figure 1E). To further identify the characteristics of monocytes/macrophages, we examined the expression of CD80 and CD86 through Western blot; the relative molecular weight was 38 and 40 kD, respectively (Figure 1F). The basal expression levels of TLR2, TLR4, CD80, and CD86 in these five types of cells were detected by Q-PCR and found that they were highly expressed in monocytes/macrophages (Figure 1E). These results indicated that our methods can successfully isolate duck neuronal, astrocytes, and monocytes/macrophages.

### Duck Primary Cells Exhibited Differing Innate Immune Responses to DNA and RNA Viruses

**Figure 2:**
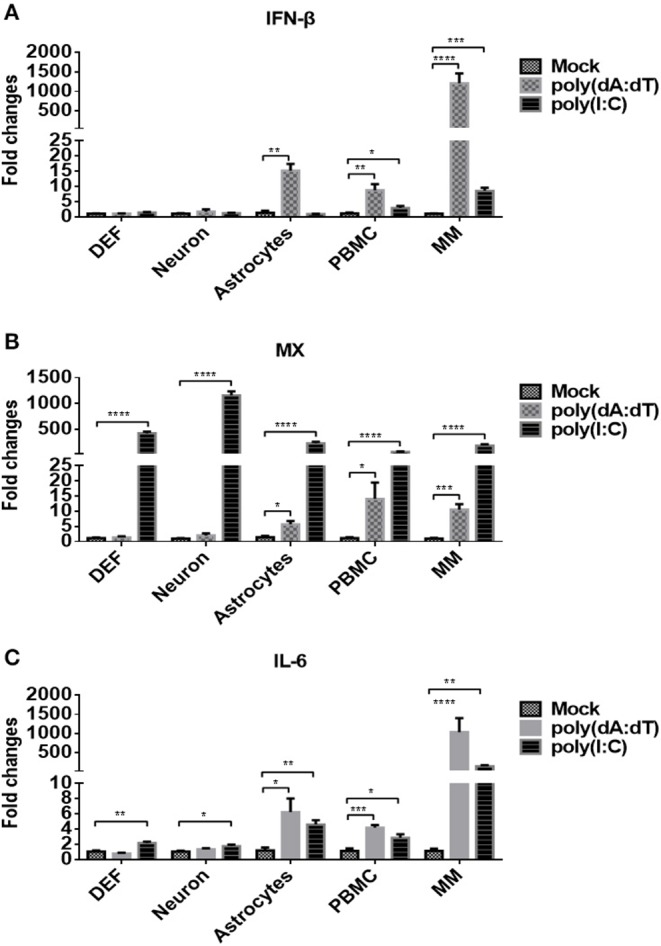
Duck DEFs, neurons, astrocytes, PBMCs, and monocytes/macrophages respond to DNA and RNA virus analogs. **(A–C)** Five types of duck primary cells were treated with the DNA virus analog poly(dA:dT) or RNA virus analogs poly(I:C) at a dose of 5 μg/ml or mock treatment for 24 h. The expression levels of IFN-β **(A)**, MX **(B)**, and IL-6 **(C)** in duck DEFs, neurons, astrocytes, PBMCs, and monocytes/macrophages were determined using Q-PCR at 24 h post-treatment. Relative expression is presented as fold change compared to mock treatment.

We then examined whether the five types of duck primary cells we isolated were able to respond to DNA and RNA viruses by stimulating the cells with the DNA and RNA virus analogs, poly(dA:dT) or poly(I:C), respectively, at a dose of 5 μg/ml. At 24 h post-treatment, the expression of IFN-β (Figure 2A) was analyzed, and the data indicated that poly(dA:dT) induced significantly higher expression of IFN-β in astrocytes, PBMCs, and monocytes/macrophages than did poly(I:C) or mock treatment. Poly(I:C) induced significantly higher levels of IFN-β expression in PBMCs and monocytes/macrophages compared with mock-treated cells.

The expression level of MX, an ISG induced by IFN or pathogens, was examined in each of the five cell types, and the data showed that poly(I:C) induced significantly higher expression of MX in all tested cells (DEFs, neurons, astrocytes, PBMCs, and monocytes/macrophages) than did poly(dA:dT) or mock treatment (Figure 2B). Under poly(dA:dT) stimulation, significant upregulation of MX expression was observed only in astrocytes, PBMCs, and monocytes/macrophages. The expression of IL-6, an inflammatory factor, was also determined after stimulation (Figure 2C), and the data showed that poly(I:C) induced IL-6 expression in all five types of duck cells. Although poly(dA:dT) induced IL-6 only in astrocytes, PBMCs, and monocytes/macrophages, the levels were higher than those induced by poly(I:C). Taken together, these data indicated that the five types of duck primary cells we examined are competent to respond to DNA and RNA viruses and that innate immune signaling is initiated. Upon stimulation with DNA viruses, monocytes/macrophages exhibit higher levels of IFN-β, ISG, and inflammatory cytokine expression than DEFs, neurons, astrocytes, and PBMCs.

### Basal Levels of Innate Immune Factors Differed in Duck Primary Cells

**Figure 3:**
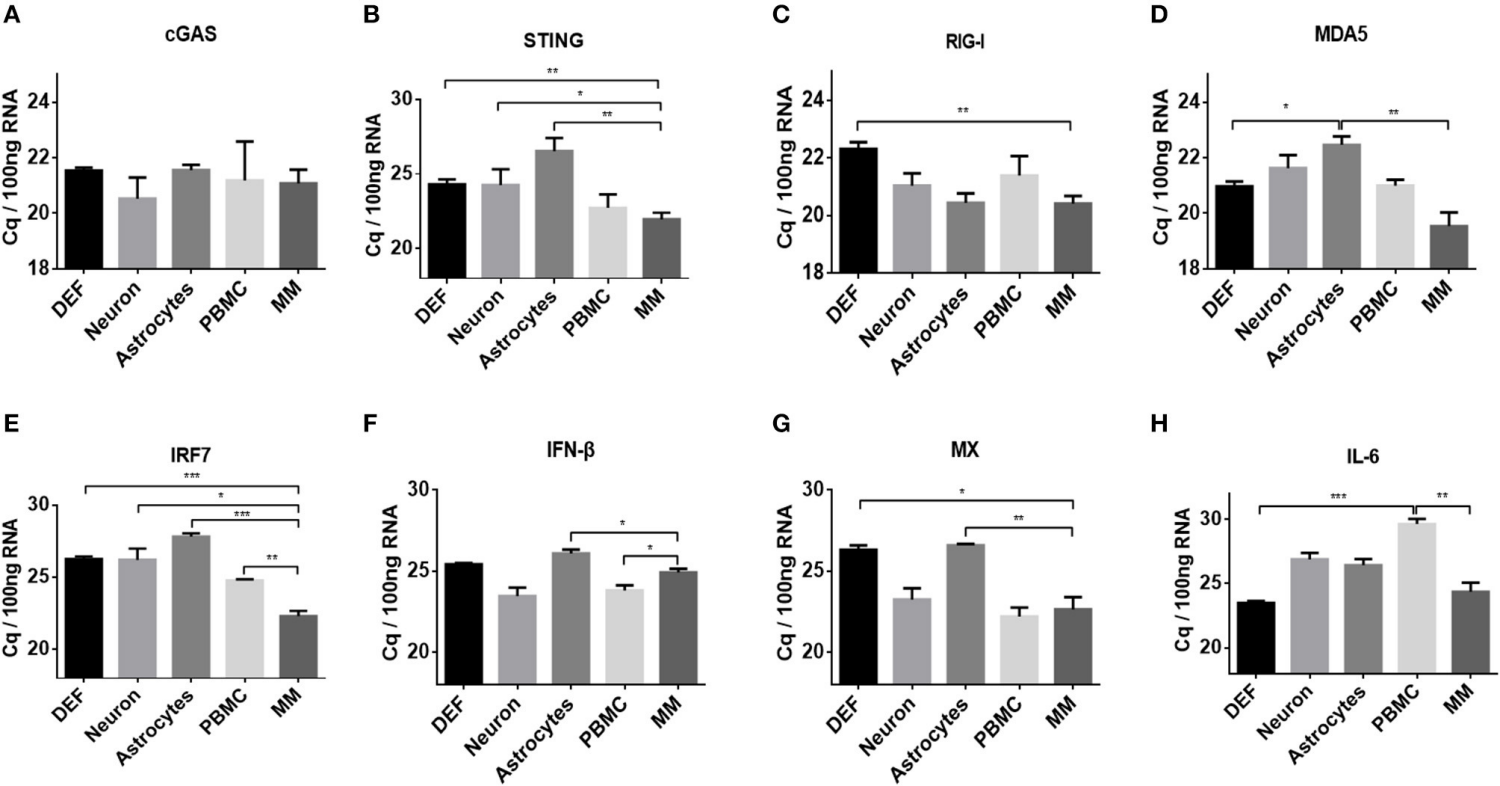
Comparison of basal levels of innate immune factors between duck DEFs, neurons, astrocytes, PBMCs, and monocytes/macrophages. **(A–H)** The basal levels of duck cGAS, STING, RIG-I, MDA5, IRF7, IFN-β, MX, and IL-6 in the five types of duck primary cells were examined using Q-PCR with specific primers. The data are presented as Cq values for 100 ng of RNA.

To elucidate the mechanisms associated with the different responses of the five types of duck primary cells to DNA and RNA virus analogs, the basal levels of innate immune factors were compared. We examined a variety of representative factors, including cGAS, STING, RIG-I, MDA5, IRF7, IFN-β, MX, and IL-6. The basal levels of cGAS were comparable among the different duck primary cells (Figure 3A). The basal levels of STING, RIG-I, MDA5, IRF7, IFN-β, MX, and IL-6 were highest in monocytes/macrophages and lowest in astrocytes and DEFs (Figures 3B–H). These results indicated that monocytes/macrophages mount a greater innate immune response than the other cell types after stimulation with DNA or RNA virus analogs.

### Duck Cells Infected With Multi-Tropic DPV

**Figure 4:**
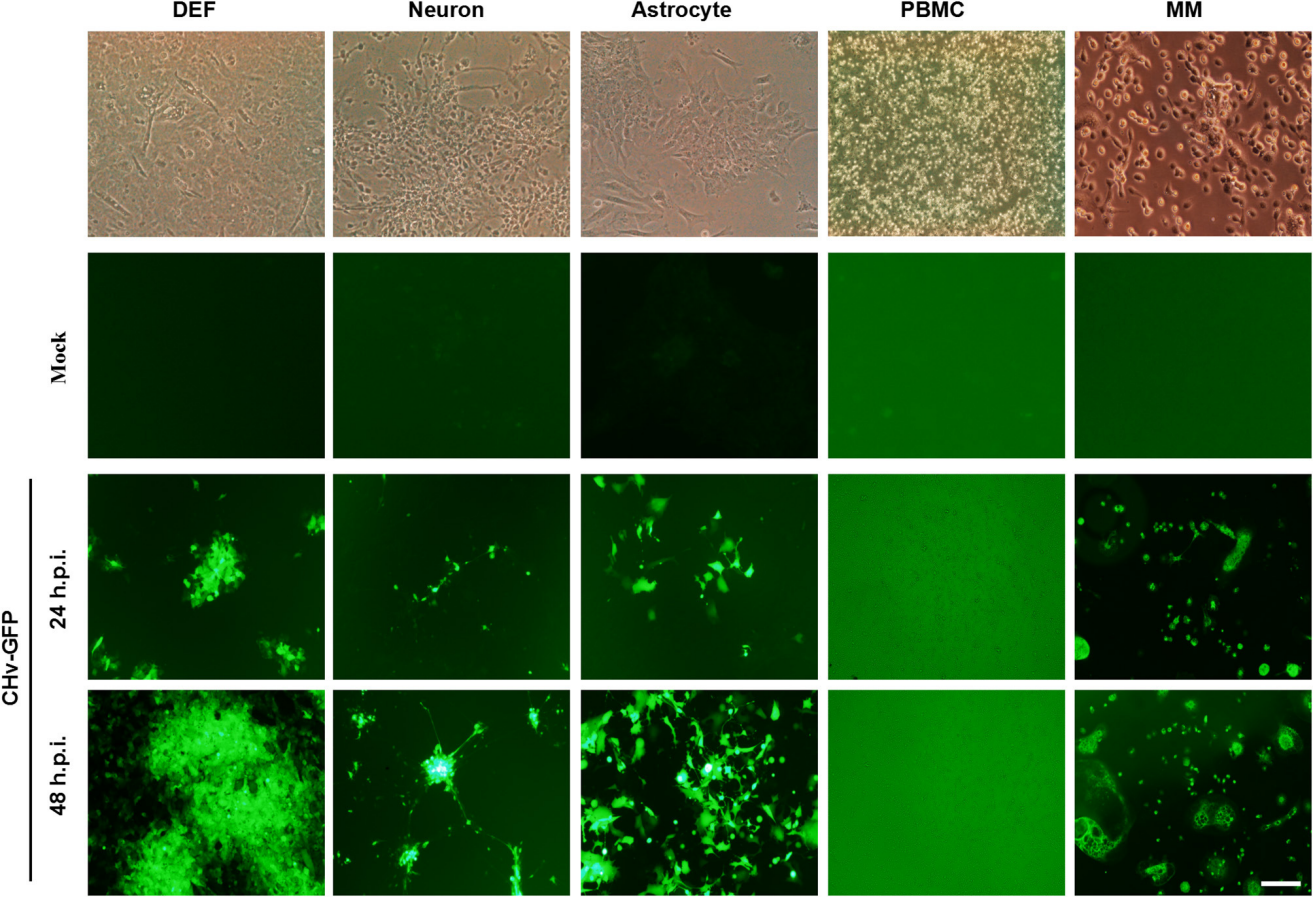
Duck cells infected with multi-tropic DPV. Duck DEFs, neurons, astrocytes, PBMCs, and monocytes/macrophages were infected with recombinant virulent strains of BAC-CHv-GFP (CHv-GFP) at a MOI of 0.01. At 24 and 48 h post-infection (hpi), viral plaques were recorded by monitoring GFP expression by fluorescence microscopy. The scale bar is 50 μm.

DPV, the only herpes virus circulating in aquatic animals, exhibits multi-tropic infection, and the virus can be detected in nearly every major organ, including the brain, lung, spleen, intestines, and liver. In order to investigate the tropism of DPV in duck cells, duck DEFs, neurons, astrocytes, PBMCs, and monocytes/macrophages were infected with a recombinant virulent virus strain, CHv-GFP, at a low MOI of 0.01 (Figure 4). Virus proliferation and morphology of the primary cells cultured *in vitro* were assessed by monitoring the expression of GFP. As the data demonstrate, at 24 h post-infection, GFP was clearly expressed in duck DEFs, neurons, astrocytes, and monocytes/macrophages, and all four types of virus-infected cells were radially enlarged at 48 h post-infection. No significant GFP expression was observed in DPV-infected PBMCs. These data demonstrated that DPV infects different types of duck cells *in vitro* and exhibits multi-tropic infection, with possible replication in many organs and tissues, such as muscle, brain (neurons and astrocytes), and spleen (monocytes/macrophages).

### Virulent and Vaccine DPV Strains Grew Differently in Duck Primary Cells

**Figure 5:**
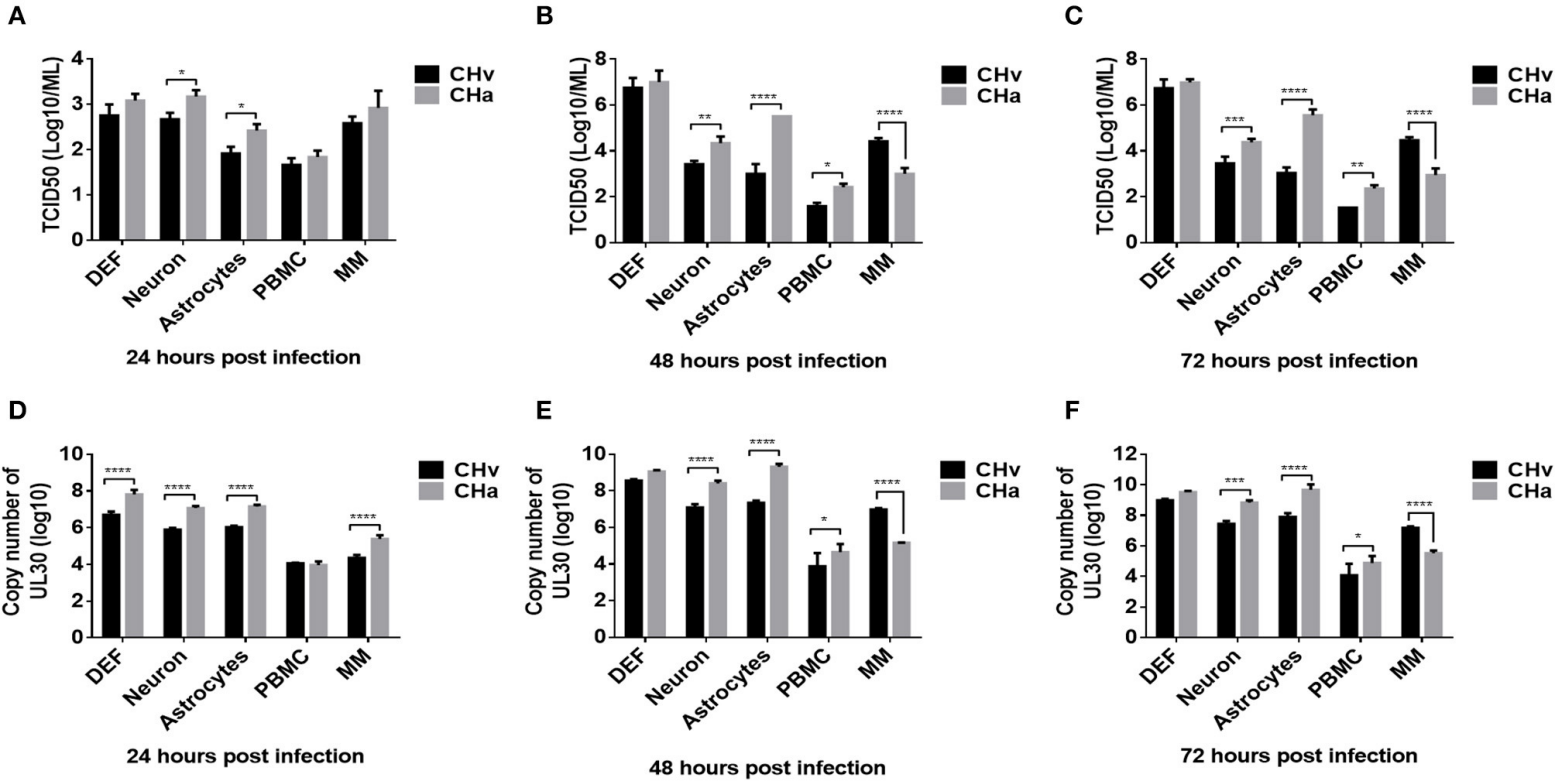
Growth dynamics of virulent and attenuated vaccine strains of DPV in duck DEFs, neurons, astrocytes, PBMCs, and monocytes/macrophages. Duck DEFs, neurons, astrocytes, PBMCs, and monocytes/macrophages were infected with DPV virulent strain, CHv strain, DPV vaccine strain, or CHa strain at a MOI of 0.01. **(A–C)** Cell culture supernatants were collected at 24, 48, and 72 h post-infection (hpi), and viral titer in the cell culture supernatants was quantified by determining the TCID_50_ of each sample. **(D–F)** Duck primary cells were collected in PBS, viral genomic DNA was extracted, and viral copy number was determined at 24, 48, and 72 hpi according to the Taqman method.

To investigate the growth dynamics of DPV in the five types of duck primary cells, the cells were infected with a virulent DPV strain (CHv) or an attenuated vaccine strain (CHa) at a MOI of 0.01. The viral titer in the cell culture supernatant was determined based on the TCID_50_ (Figures 5A–C), and the intracellular viral genome copy number was also determined (Figures 5D–F). CHa produced higher virus titer and genomic copy number than CHv in neurons and astrocytes at 24, 48, and 72 hpi. The viral titer and genome copy number of CHa were comparable to CHv at 24 hpi but higher than CHv at 48 and 72 hpi in PBMCs, although the values were near the detection limit. CHa produced higher viral genome copy number in DEFs at 24 hpi (Figure 5D), but the viral titer was comparable at 24, 48, and 72 hpi (Figures 5A–C). These data indicated that the attenuated strain of DPV replicates faster and produces more virus particles than the virulent strain in duck DEFs, neurons, astrocytes, and PBMCs.

An intriguing finding was that the titers of both virus strains were comparable in monocytes/macrophages at 24 hpi (Figure 5A). The genomic copy number of CHa was significantly higher than that of CHv at 24 hpi (Figure 5D). The genomic copy number and viral titer of CHa slowly decreased between 48 and 72 hpi. In contrast, the genomic copy number and viral titer of CHv increased over this time period (Figures 5B,C,E,F). The copy number and viral titer of CHa was significantly lower at 48 and 72 hpi compared with CHv (Figures 5B,C,E,F). These data demonstrate that the attenuated strain (CHa) abortively infects duck monocytes/macrophages, whereas the virulent strain (CHv) persistently infects duck monocytes/macrophages, we speculate that the monocytes/macrophages may be able to kill the virus and it is an active immune response that is responsible for the decrease in viral titer of CHa strain, as we demonstrated in the experiments below.

### The Innate Immune Response Was Inhibited by the Virulent DPV Strain but Activated by the Attenuated DPV Strain

To determine whether the different growth dynamics exhibited by the virulent and attenuated strains of DPV affected the innate immune response in duck primary cells, the expression levels of cGAS, STING, RIG-I, MDA5, IRF7, IFN-β, MX, and IL-6 were analyzed in the five types of duck primary cells infected with either the CHv strain or CHa strain at a MOI of 1.0. There were no significant differences in the expression of these molecules in the CHv- or CHa-infected DEFs or neurons at 6, 12, and 24 hpi, but there was a slight increase in expression in astrocytes infected with CHa (data did not show). In PBMCs, infection with the CHv strain induced cGAS, STING, MDA5, IRF7, IFN-β, MX, and IL-6 expression early, at 6 hpi, but expression decreased by 12 and 24 hpi (data did not show). Infection with the CHa strain induced a slight increase in the expression levels of these factors at 6 hpi, and the expression continued to increase with time and was significantly higher compared with CHv-infected cells or mock-treated cells. However, the CHv strain may not be able to infect PBMCs observed from Figures 4, 5, and the virus level of the CHa strain infected with PBMCs is slightly higher than that of the CHv strain, so comparing the innate immune responses caused by the two in PBMCs are complicated.

**Figure 6:**
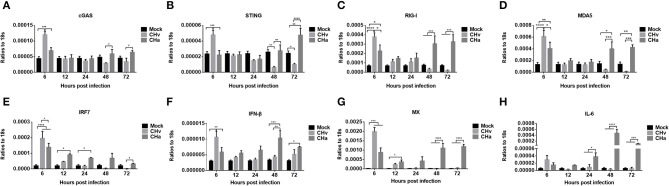
The innate immune response in monocytes/macrophages was repressed by the virulent strain of DPV and activated by the attenuated strain at later time points. **(A–H)** Monocytes/macrophages were differentiated from PBMCs induced with human M-CSF at a concentration of 50 ng/ml for 7 days and then infected with either the CHv or CHa strain at a MOI of 1.0. At various time points after infection, the expression levels of cGAS, STING, RIG-I, MDA5, IRF7, IFN-β, MX, and IL-6 were determined using Q-PCR with specific primers. The data are presented as the ratio to 18s RNA in each sample.

The expression levels of cGAS, STING, RIG-I, MDA5, IRF7, IFN- IFN-β, MX, and IL-6 were also examined in CHv- and CHa-infected duck monocytes/macrophages. Similar to the changes observed in DPV-infected PBMCs, infection with CHv induced significant increases in the expression of cGAS, STING, RIG-I, MDA5, IRF7, IFN-β, and MX in monocytes/macrophages early, at 6 hpi, but expression rapidly decreased by 12, 24, 48, and 72 hpi (Figure 6), a trend opposite to the growth curve on monocytes/macrophages (Figure 5). Infection with the CHa strain induced significantly increased expression of cGAS, RIG-I, MDA5, IRF7, IFN-β, and MX in monocytes/macrophages at 6 hpi, and these levels were higher than those observed in mock-treated cells but lower than those in cells infected with CHv strain. The expression of cGAS, STING, RIG-I, MDA5, IRF7, IFN-β, MX, and IL-6 decreased slightly at 12 and 24 hpi, but then increased markedly at 48 and 72 hpi (Figure 6). These data indicated that the virulent DPV strain activates the innate immune system in PBMCs and monocytes/macrophages during the early stages of infection, but then the innate immune response is downregulated once the virus begins to replicate. The attenuated strain (CHa) activates the innate immune system primarily during the later stages of infection, and this could be the reason why this strain abortively infects monocytes/macrophages and has been attenuated.

### Blockage of IFNAR Signaling Enhanced CHa Strain Replication in Astrocytes, PBMCs, and Monocytes/Macrophages

**Figure 7:**
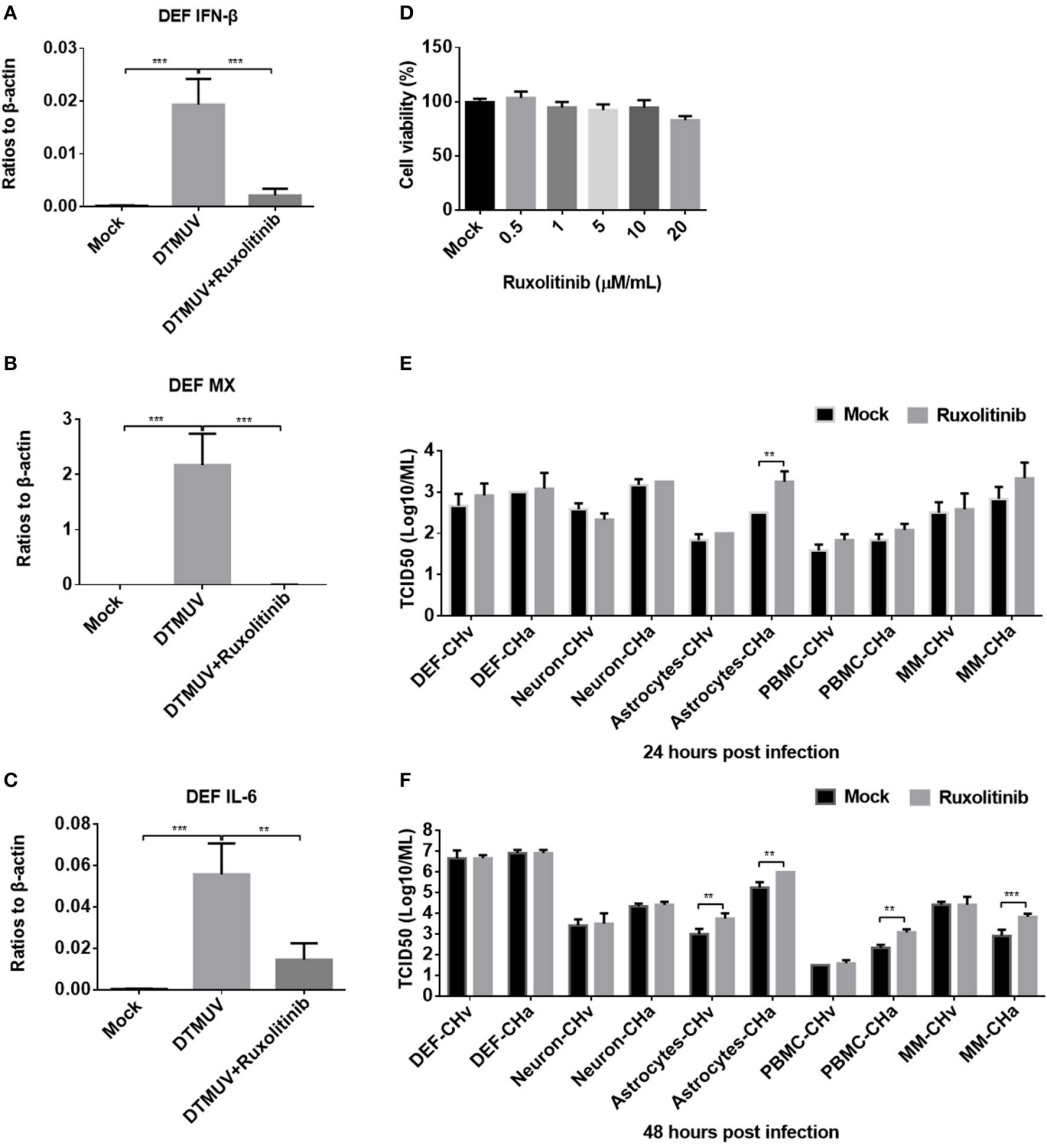
Blockage of IFNAR signaling enhanced replication of the attenuated DPV strain (CHa) in astrocytes, PBMCs, and monocytes/macrophages. **(A–C)** DEFs were pretreated with the IFNAR inhibitor ruxolitinib at a concentration of 5 μM for 1 h and then infected with DTMUV at a MOI of 0.1. After infection, the inhibitor was kept at the same concentration for 24 h. The expression levels of IFN-β, MX, and IL-6 were determined by Q-PCR at 24 h post-infection. The data are presented as the ratio to β-actin. **(D)** The DEF cell was treated with ruxolitinib at a dose of 0.5, 1, 5, 10, and 20 μM/ml for 24 h; DMSO was added in the mock group for control, and then the cell viability was tested using MTT method. The results were presented as percentages (%) to mock. **(E,F)** Duck DEFs, neurons, astrocytes, PBMCs, and monocytes/macrophages were pretreated with ruxolitinib at a concentration of 5 μM/ml for 1 h and then infected with either the CHv or CHa strain at a MOI of 0.1. After infection, the inhibitor was kept at the same concentration. The cell culture supernatants were collected at 24 and 48 h post-infection, and the viral titers were determined in the cell culture supernatant based on the TCID_50_.

To further investigate how DPV infection affects the innate immune response in the five types of duck primary cells, IFNAR signaling was blocked using the specific inhibitor ruxolitinib. We first examined whether ruxolitinib functions in the duck cells by pretreating DEFs with ruxolitinib for 1 h and then infecting the cells with a duck RNA virus, DTMUV, which belongs to the Flavivirus family and was demonstrated to induce strong innate immune response in DEF cells (30). After infection, rucolitinib was added at the same concentration and the cells were incubated for 24 h. DTMUV induced significant expression of IFN-β, MX, and IL-6 in DEFs (Figures 7A–C), but the expression levels of these factors were clearly reduced in cells treated with ruxolitinib, indicating that this inhibitor can be used to block IFNAR signaling in duck cells.

The cell toxicity of ruxolitinib on DEFs (Figure 7D), neurons, astrocytes, PBMCs, and monocytes/macrophages was determined (data did not show), and no obvious cell viability was changed by ruxolitinib at a dose of 5 μM/ml on these five cell types. Ruxolitinib was used to treat duck DEFs, neurons, astrocytes, PBMCs, and monocytes/macrophages in further assays. Viral titer was examined at various time points in the culture supernatants of cells infected with either the CHv or CHa DPV strain, with and without ruxolitinib treatment. After ruxolitinib treatment for 24 hpi, the titer of CHa in the astrocyte culture supernatant was significantly increased, by ~10-fold; however, at this time point, there were no obvious changes in CHa titer in the cell culture supernatants of DEFs, neurons, PBMCs, or monocytes/macrophages (Figure 7E). At 24 hpi, no change in CHv titer was detected in any of the five duck primary cells (Figure 7E). At 48 hpi, the titer of CHa was significantly increased in the cell culture supernatants of astrocytes, PBMCs, and monocytes/macrophages (Figure 7F). There was a certain increase observed in the culture supernatant of astrocytes infected with CHv. No changes were observed in the titers of either CHv or CHa in the supernatants of neurons and DEFs treated with ruxolitinib (Figures 7E,F). Taken together, these data indicated that blockage of IFNAR signaling enhances the replication of the attenuated DPV strain (CHa) in duck astrocytes, PBMCs, and monocytes/macrophages. Infection with CHa activates the type I IFN response in these cells to a greater degree than does infection with the CHv strain.

### Priming Monocytes/Macrophages With an Agonist Restricted CHv Replication

**Figure 8:**
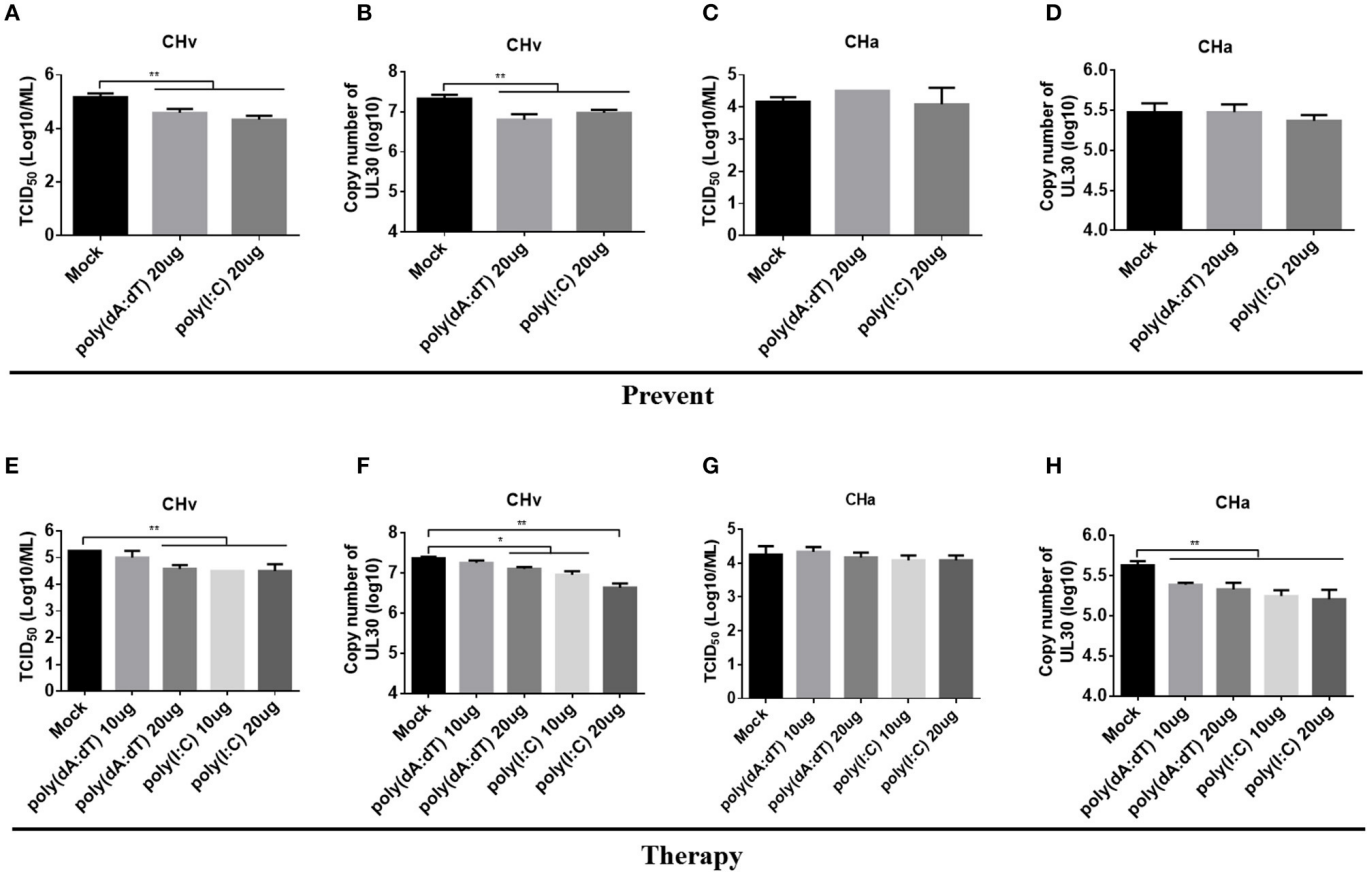
Activation of IFNAR signaling reduced replication of the virulent strain (CHv) in monocytes/macrophages. **(A–D)** Duck monocytes/macrophages (MM) were pretreated with poly(dA:dT) or poly(I:C) at a concentration of 20 μg/ml for 12 h and then infected with either the CHv or CHa strain at a MOI of 1.0. At 24 h post-infection, the cell culture supernatants were collected for TCID_50_ determination **(A,C)**, and the cells were washed with PBS, removed by scraping, and collected for determination of viral genomic copy number **(B,D)**. **(E–H)** Duck monocytes/macrophages were pretreated with poly(dA:dT) or poly(I:C) at a concentration of 10 or 20 μg/ml for 2 h and then infected with either the CHv or CHa strain at a MOI of 1.0. After infection, the analogs were added at the same concentration and incubated for 24 h. At 24 h post-infection, the cell culture supernatants were collected for TCID_50_ determination **(E,G)**, and the cells were washed with PBS, removed by scraping, and collected for determination of viral genomic copy number **(F,H)**.

Infection with the CHv or CHa strains of DPV had a differential effect on the innate immune response in duck primary cells. Both strains of DPV induced the most significant changes in the innate immune response in monocytes/macrophages (Figure 6), and therefore monocytes/macrophages were chosen as a cell model to investigate the antiviral role of IFNAR signaling. Monocytes/macrophages were pretreated with poly(dA:dT) or poly(I:C) at a dose of 20 μg/ml for 12 h and then infected with the CHv or CHa strain at a MOI of 1.0. The viral titer and genomic copy number were determined at 24 hpi. The viral titer and genomic copy number of the CHv strain were significantly decreased in monocytes/macrophages pretreated with poly(dA:dT) or poly(I:C) (Figures 8A,B), but there was no change in either viral titer or genomic copy number of the CHa strain (Figures 8C,D).

To assess the therapeutic antiviral effect of the agonists, monocytes/macrophages were pretreated with poly(dA:dT) or poly(I:C) at dose of 10 or 20 μg/ml for 2 h and then infected with either the CHv or CHa strain at a MOI of 1.0. After infection, the same concentration of poly(dA:dT) or poly(I:C) was added and the cells were incubated until the time point of examination. At 24 hpi, the viral titer and genomic copy number were determined. As the data showed, treating monocytes/macrophages with poly(dA:dT) at a dose of 10 μg/ml had no effect on viral titer or copy number of the CHv strain, but a significant reduction of viral titer and genomic copy number was observed at a dose of 20 μg/ml (Figures 8E,F). Significant reductions in both viral titer and genomic copy number of the CHv strain were observed with monocytes/macrophages treated with poly(I:C) at a dose of either 10 or 20 μg/ml (Figures 8E,F). For monocytes/macrophages infected with the CHa strain, treatment with poly(dA:dT) or poly(I:C) at a dose of either 10 or 20 μg/ml resulted in a reduction in viral genome copy number (Figure 8H) but had no effect on viral titer (Figure 8G). Taken together, these data indicate that activation of IFNAR signaling via a PRR agonist restricts the virulent strain of DPV. PRR agonists thus exhibit preventative and therapeutic potential against DPV infection.

## Discussion

In the current study, we isolated and cultured duck primary DEFs, neurons, astrocytes, PBMCs, and monocytes/macrophages. To our knowledge, this is the first report of the culture of duck neurons, astrocytes, and monocytes/macrophages *in vitro*. These five types of duck primary cells were used to investigate the tropism of DPV and the innate immune response to DPV infection. We found that DPV persistently infected and replicated in DEFs, neurons, astrocytes, and monocytes/macrophages, indicating that DPV exhibits wide *in vivo* organ tropism in ducks (19). Indicative of the potential of DPV to evade the innate immune response, and infection of DEFs, neurons, and astrocytes, there were slight changes in PRR and ISG expression. Upon infection of PBMCs and monocytes/macrophages at a high MOI, the CHv strain only upregulated the expression of some PRRs and ISGs at 6 hpi, but expression of these innate immunity factors was downregulated at later time points by the virulent DPV strain. A particularly intriguing finding was that the expression of PRRs and ISGs in PBMCs and monocytes/macrophages was consistently upregulated by infection with the attenuated vaccine strain (CHa). Monocytes/macrophages represent an ideal cell model for investigating the relationship between DPV infection and the innate immune response; as the virulent strain of DPV persistently infects monocytes/macrophages, these cells may play a critical role in the pathogenicity of the virus.

The current lack of methods for culturing duck primary cells significantly hinders investigations of the role of each cell type in the infectious cycle, tropism, and pathogenicity of aquatic bird viruses, such as DPV, avian influenza virus, novel duck reovirus (NDRV), and DTMUV, which cause major economic losses in the duck industry (34–37). As opposed to RNA viruses such as avian influenza, NDRV, and DTMUV, DPV is a dsDNA virus that does not readily induce an innate immune response in DEFs. Hence, in order to investigate the mechanism by which the virus evades the innate immune response, a better primary cell model is needed. Numerous types of mammalian primary cells have been isolated from the tissues and successfully cultured, but there are few such reports regarding the culture of primary cells of aquatic animals such as ducks and geese. In the present study, we isolated and cultured duck neurons, astrocytes, and monocytes/macrophages using methods developed for mouse or human cells. Our data indicated that the synapses of neurons, the classic morphology, formed at 3 days post-induction. The expected star shape was clearly observed for duck astrocytes isolated from brain tissue and cultured in DMEM containing 10% FBS. It was surprising that we could induce the differentiation of wheel-shaped monocytes/macrophages from duck PBMCs using human or mouse M-CSF at a concentration of 50 ng/ml, and no living cells were detected at 7 days post-seeding in the absence of human or mouse M-CSF.

As a herpesvirus, DPV sometimes exhibits chronic or latent infection in the duck trigeminal ganglion (TG) (38), similar to HSV-1 (39). Studies of the molecular mechanism of HSV-1 latency typically use mouse or rabbit animal models, but not humans. However, the research using an *in vitro* latency model in neurons would be a preferable approach prior to *in vivo* studies. Infection of stem-derived neurons with a low viral dose of wild-type HSV-1 in the presence of the antiviral agent acyclovir and interferon-alpha results in the establishment of a latent, non-productive infection. In this state, viral replication and expression of late viral gene markers are not detected, but there is an accumulation of viral latency-associated transcript RNA (40). In our current research, we isolated and cultured duck neurons, and both the virulent and attenuated strains of DPV were able to infect neurons in the absence of treatment with any antiviral drugs. This would be a good cell model for further studies of the molecular mechanism underlying latent DPV infection in neurons or investigations of how DPV invades the CNS to cause fever and intracranial swelling.

Astrocytes are basal, functional cells of the CNS that form and maintain the permeability of the blood–brain barrier and restrict pathogen invasion (41, 42). The TLR3 in astrocytes plays a critical role in containing the replication and transmission of HSV-1 in the CNS; deficiency of TLR3 causes astrocytes to become permissive to HSV-1 infection, thus facilitating infection of the entire CNS by HSV-1 (43). IFNAR signaling in astrocytes exhibits regional differences that act to restrict the invasion of neurotropic viruses; loss of IFNAR signaling decreases the survival of mice after West Nile virus infection (44). In our present study, we isolated and cultured duck astrocytes. The basal levels of innate immune factors in astrocytes were the lowest among the five types of duck primary cells we examined. Both strains of DPV infected astrocytes, and the titer of the attenuated strain (CHa) in astrocytes was comparable to that in DEFs. Blockage of IFNAR signaling increased DPV replication in astrocytes. Thus, astrocytes may play a critical role in containing DPV infection in the CNS and might therefore be a useful model. Further investigations of the role of astrocytes in DPV invasion and latent infection in the CNS are warranted.

Persistent infection with HSV-1 is a prerequisite to this virus's pathogenicity, and HSV-1 has been reported to infect sensory neurons, the corneal epithelium, lymphocytes, and macrophages (45). In the sensory ganglia, macrophages infiltrate the TG and produce TNF-α and iNOS to control the primary HSV-1 infection (46). In human primary macrophages, knockdown of MDA5 and MAVS strongly inhibits the expression of IFN and TNF-α induced by HSV-1 entry and replication, indicating that the early innate recognition of HSV-1 involves MDA-/MAVS-dependent pathways (47). The expression of chemokines such as CXCL10 and CCL3 in human monocyte-derived macrophages induced by HSV-1 infection involves IFI16-dependent and IFI16-independent pathways (48). Activation of IFNAR signaling promotes high ISGs expression in macrophages infected with HSV-1, in which SAMHD1 inhibits HSV-1 propagation by limiting viral DNA synthesis (49). Abortive infection of viruses in astrocytes or macrophages appears to be essential to induce innate and adaptive immune responses to restrict or clear vesicular stomatitis virus, rabies virus, or influenza virus (29, 50, 51). In our current study, monocytes/macrophages were infected by both strains of DPV, and the viral titer and copy number of the virulent strain (CHv) increased from 24 to 72 hpi at a MOI of 0.01. In contrast, the viral titer and genomic copy number of the attenuated strain (CHa) began to decrease early in the infection (Figure 5). From these data, we inferred that the virulent strain (CHv) persistently infects the monocytes/macrophages, whereas the attenuated strain (CHa) causes an abortive infection in monocytes/macrophages. We further verified these observations by examining the innate immune response induced by these two strains. In duck monocytes/macrophages, the expression of PRRs and ISGs were induced by both strains at 6 hpi but gradually decreased in CHv-infected cells after 6 hpi (Figure 6). However, in CHa-infected monocytes/macrophages, the expression of PRRs and ISGs were induced at 6 hpi and declined slightly at 12 and 24 hpi before rapidly increasing between 48 and 72 hpi (Figure 6). In the later time points of infection, the titer of the CHa strain was significantly lower than that of the CHv strain (Figure 5). In the previous studies on DPV and MDV viruses, researchers observed the slight up-regulation of the innate immune molecules around 6 hpi in the fibroblasts, and the rapid down-regulation of these molecules from 12 hpi (23, 52). These results are consistent with the data we obtained in this study; we hypothesized that DPV virulent strains have multiple, highly potent proteins that inhibit innate immunity, and the mechanism of suppressing innate immunity will be further studied in the future.

Blockage of IFNAR signaling in monocytes/macrophages led to a marked increase in the titer of the CHa strain, but not that of the CHv strain (Figure 7). Priming monocytes/macrophages with poly(dA:dT) or poly(I:C) selectively restricted the replication of the CHv strain (Figure 8). The growth dynamics and innate immune response were similar in PBMCs. These data indicated that the CHv and CHa strains exhibit different replication patterns in monocytes/macrophages based on activation or inhibition of the innate immune response. This could explain why the CHa strain has been attenuated and provides protection from infection with the virulent DPV strain in ducklings. Monocytes/macrophages thus represent a promising cell model suitable for further studies of DPV pathogenicity and the mechanism of innate immune response evasion by DPV. In a recent study, the authors found that monocytes/macrophages are important target cells for DTMUV infection (53). DTMUV successfully infects macrophages by subverting the innate immunity of monocytes/macrophages and transmits in the body using monocytes/macrophages as cell carriers. In that study, the authors separated lymphocytes and monocytes/macrophages by washing the PBMCs at 2 and 24 h after plating and then used the cells for assays. Though the detection with antibodies against the hypothetical duck CD68 protein showed that the purity of monocytes/macrophages were greater than 85%, we think that it is difficult to obtain large numbers of macrophages from PBMCs if the monocytes are not induced with M-CSF or GM-CSF. Therefore, the duck monocytes/macrophages isolated in our study will provide an important cell manipulation platform for similar researches and obtain more direct experimental results and conclusions.

In conclusion, we isolated and cultured primary duck DEFs, neurons, astrocytes, PBMCs, and monocytes/macrophages. These five types of duck primary cells were able to respond to DNA and RNA virus stimulators and exhibited upregulated expression of PRRs and ISGs, thus demonstrating that they are useful cell models for deeper investigations of the induction/evasion of the innate immune response by aquatic animal viruses such as DPV. The virulent and attenuated strains of DPV infected these cells with differential replication dynamics, accompanied by differences in the innate immune response. Blockage of IFNAR signaling enhanced the replication of the attenuated strain of DPV in astrocytes, PBMCs, and monocytes/macrophages. Priming the IFNAR signaling pathway specifically reduced the titer of the virulent strain of DPV, indicating that the IFNAR signaling pathway plays a key role in limiting DPV infection. Persistent infection of monocytes/macrophages coupled with inhibition of the innate immune response represents an important junction in the pathogenicity of DPV.

## Data Availability Statement

All datasets generated for this study are included in the article/supplementary material.

## Author Contributions

BT and MW: conceived and designed the experiments. DC, TH, LW, LD, YW, XZ, and ML: performed the experiments. BT, SC, JH, QY, RJ, DZ, XO, SM, YY, YL, LZ, and SZ: analyzed the data. BT, DC, MW, and AC: wrote the paper.

### Conflict of Interest

The authors declare that the research was conducted in the absence of any commercial or financial relationships that could be construed as a potential conflict of interest.
